# Risk and Protective Factors Related to Menopause, Women’s Healthy Aging, and Cognition

**DOI:** 10.1093/geroni/igaf122.1800

**Published:** 2025-12-31

**Authors:** Amber Watts, Amy Costa, Amber Watts

## Abstract

About 1.3 million women per year in the U. S. experience the menopause transition. Given modern life expectancies, women spend more than one-third of their lifetime post-menopause. The menopause transition is a critical window for positive and negative influences on women’s healthy aging and cognition. This symposium proposes to bring together research from five laboratories investigating risk and protective factors for menopause health and women’s healthy aging including lifestyle (sleep, exercise), environment (pollutants, greenspace), and psychological risks (childhood adversity, depression) that occur throughout women’s lifelong development. The projects present a range of methodologies including clinical intervention, cognitive and brain health assessment, survey design, and narrative review. Results of these projects demonstrate that aging trajectories differ by sex and that characteristics of the menopause transition are important for physical, psychological, and cognitive health outcomes related to aging. Results inform the development of effective interventions for healthy menopause and aging in women. They add to theoretical knowledge regarding the key contributors to healthy menopause and aging in women. Additionally, this symposium provides an opportunity for junior scholars (pre-doctoral, early career) and senior scholars (mid- to late-career) to connect to advance scholarship and to build representation of menopause research at GSA, a topic that has recently been under the public spotlight in news media, as well as privately and federally funded research. Women’s Issues Interest Group Sponsored Symposium

